# A Case Report of Iatrogenic Pulmonary Artery Injury due to Chest-Tube Insertion Repaired under Cardiopulmonary Bypass

**DOI:** 10.1155/2013/590971

**Published:** 2013-09-11

**Authors:** Ciss Amadou Gabriel, Dieng Papa Adama, Ba Papa Salmane, Gaye Magaye, Diatta Souleymane, Leye Mohamed, Fall Lamine, Sene Etienne Birame, N'diaye Assane, Diarra Oumar, Kane Oumar, N'diaye Mouhamadou

**Affiliations:** ^1^Thoracic and Cardiovascular Surgery, University Hospital of Fann, CHN Fann Dakar Sénégal, BP 5035, Dakar, Senegal; ^2^Cardiology, University Hospital of Fann, CHN Fann Dakar Sénégal, BP 5035, Dakar, Senegal; ^3^Intensive Care Unit, University Hospital of Fann, CHN Fann Dakar Sénégal, BP 5035, Dakar, Senegal

## Abstract

The authors presented a case of a 50-year-old patient with multiple trauma who suffered from the inadvertent cannulation of the main pulmonary artery at the second attempt of left chest drainage. Pulmonary artery injury has been suspected because early chest tube production was 2300 mL of blood. CT scan showed injury of the trunk of the pulmonary artery, left hemothorax, and suspect damage of the right branch of the pulmonary artery. That chest tube touched the posterior wall of ascending aorta. Surgical approach was median sternotomy. Exploration showed a perforation of the trunk of pulmonary artery without lesion of the right pulmonary branch and the posterior wall of the ascending aorta. The lesion was repaired under normothermic partial cardiopulmonary bypass. Postoperative period was free of events. Review of the literatures for this rare case report has been done.

## 1. Case Presentation

The authors report a case of a 50-year-old patient with multiple trauma due to direct impact in car crash.

He presented with multiple costal fractures from 9th to 12th ribs, a diaphragm rupture with lung contusion and moderate left hemothorax. He also presented with abdominal contusion and stable fracture of the right pubic ramus without urinary disorder.

**Figure 1:**
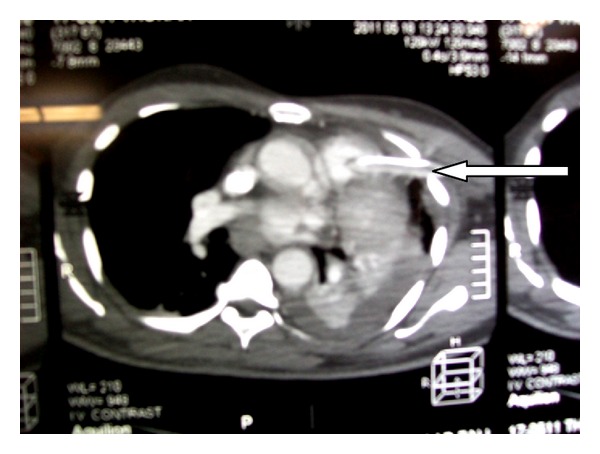
Chest tube inside pulmonary artery.

**Figure 2:**
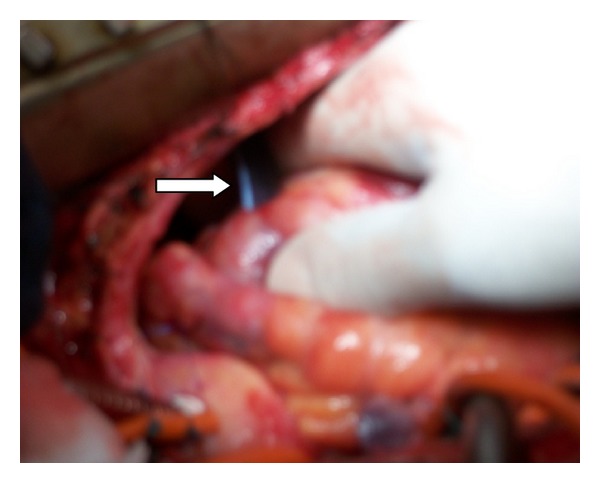
Operative view after cardiopulmonary bypass started.

The hemodynamic status was stable. Thoracoabdominal CT scan then laparotomy confirmed the diagnosis of 12 cm diaphragmatic rupture at the left side admitting a stomacal hernia. A section of the mesenteric root and an injury of the spleen capsule without active bleeding were observed. The diaphragmatic injury was sutured after fixation of the mesenteric lesion and stomacal reposition. A 32 Fr chest tube was placed through the left 5th intercostal space for drainage. Postoperative period was eventless in this conscious patient. After 48 hours in the Intensive Care Unit, the chest tube was removed, but 3 days later X-ray showed a recurrence of the hemothorax. A new tube drainage was decided. A 32 Fr chest tube was placed through the 3rd intercostal space in the anterior axillary line. The early production was 2300 mL of blood while the patient experienced a cardiovascular collapse. The tube was immediately clamped. The drop of the hemoglobin level to 4 g/dL indicated a transfusion of 2 blood bags iso rhesus (1800 mL). The patient benefited from central venous catheter placement, arterial catheter placement, and orotracheal intubation. The CT scan showed an injury of the trunk of pulmonary artery due to the chest tube (Figure 1); the tip of the tube was located inside the lumen of the right pulmonary artery next to the posterior wall of the ascending aorta. This patient with stable hemodynamic status was admitted in such condition in our facility. Surgical exploration was decided with sternotomy access. A complete median sternotomy permitted to see a lateral wound of the trunk of pulmonary arteries, and the tube was touchable over the right pulmonary artery wall (Figure 2), without lesion of the posterior wall of the ascending aorta. He also had left pleural lesion and massive hemothorax without lung lesion.

A normothermic partial cardiopulmonary bypass was settled for assistance with an aortic cannula and 2 venous cannulas. After half of the theoretical patient's cardiac flow (2.1 liters) pumped, the pulmonary artery was exposed with a tissue placed under the left ventricle next to the posterior pericardium wall. After drainage of the heart, the pulmonary artery trunk was explored as well the right pulmonary artery and the posterior wall of the ascending aorta, without discovering any other injuries. The defect was closed using a polypropylene stitch and Teflon-reinforced mattress sutures. After complete hemostasis, the cardiopulmonary bypass was stopped. The sternum was closed after irrigation of the pericardium and placement of 3 tubes for drainage (one tube in the left pleural). Postoperative period was free of events in the Intensive Care Unit, and the patient was discharged after tubes removal. On the seventh postoperative day a CT scan showed normal pulmonary artery with its wall being in good shape. Repeated CT scans at 1 month and 3 months remained normal.

## 2. Discussion

Chest tube drainage is largely used in thoracic surgeries, and the intensive care units. Such procedure is used for the drainage of air and fluid (hemothorax, pyothorax, chylothorax). Some complications could occur after chest tube drainage: hemorrhage due to intercostal artery injury and visceral injury (lung, heart, diaphragm, and abdomen). Pulmonary artery injury is a rare emergency situation. The pulmonary artery lesions are frequent in patients with pleuropulmonary adherences [1, 2]. The tube can easily penetrate the lung, injuring the pulmonary artery or their branches. Bleeding into the tube was suspected as an artery injury. Emergent tube clamping is helpful for hemostasis and life salvage. Surgical care has two objectives: hemostasis and artery injury repair. Surgical access is thoracotomy which allows good repair in case of single pulmonary artery injury. That access is direct to the pulmonary artery. When the repair is impossible, a pneumonectomy could be an alternative [3]. This access has 2 problems: access is difficult (small room view) and the hemostasis control is challenging; because of such issues, pneumonectomy is often used as a final treatment. Sternotomy access and normothermic partial cardiopulmonary bypass for cardiac assistance without aorta clamping allow drainage of heart cavities, good perfusion of the body, and good exposure of the pulmonary artery as well as the aorta wall. Also blood saving is significant. In this emergency situation, the chest tube must be clamped and must not be removed until the hemostasis control is achieved. Some authors suggest progressive occlusion of the tube, but such option could lead to extension of the clot into the pulmonary artery [4]. This option should be limited to patients with heavy pleuropulmonary adherences, nonfunctional lung, and high risk patients.

## 3. Conclusion

Pulmonary artery injury during chest tube drainage is a rare complication but a serious one. Surgical care under normothermic partial cardiopulmonary bypass allows anatomic repair with low morbidity. 

## References

[1] Takanami I (2005). Pulmonary artery perforation by a tube thoracostomy. *Interactive Cardiovascular and Thoracic Surgery*.

[2] Kao C-L, Lu M-S, Chang J-P (2007). Successful management of pulmonary artery perforation after chest tube insertion. *Journal of Trauma*.

[3] Rombolá CA, Tomatis SB, Honguero Martínez AF, Atance PL (2008). Parapneumonic pleural effusion. Accidental insertion of a chest tube into right pulmonary artery. *European Journal of Cardio-thoracic Surgery*.

[4] Sundaramurthy SR, Moshinsky RA, Smith JA (2009). Non-operative management of tube thoracostomy induced pulmonary artery injury. *Interactive Cardiovascular and Thoracic Surgery*.

